# Supplementary figures and images for: SUNi mutagenesis: Scalable and uniform nicking for efficient generation of variant libraries
Source: PLoS One. 2023 Jul 7;18(7):e0288158. doi: 10.1371/journal.pone.0288158 (PMC10328370; doi:10.1371/journal.pone.0288158)

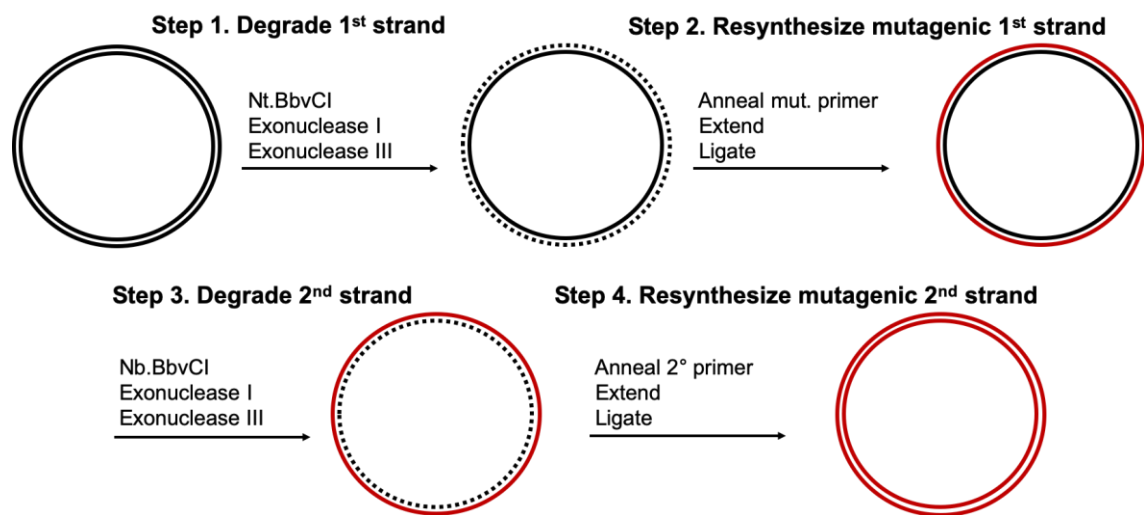

**Supplementary Figure 1. Schematic overview of the four main steps of nicking mutagenesis**

Supplement: S1 Fig — (PDF) [file pone.0288158.s001.pdf]
