# Supplementary material for: SUNi mutagenesis: Scalable and uniform nicking for efficient generation of variant libraries
Source: PLoS One. 2023 Jul 7;18(7):e0288158. doi: 10.1371/journal.pone.0288158 (PMC10328370; doi:10.1371/journal.pone.0288158)
Supplement: S2 Fig — Plasmids with either one or two BbvCI sites were digested with Nt.BbvCI, exonuclease I and exonuclease III, per the original nicking protocol, and digestion products were visualized on an agarose gel. (PDF) [file pone.0288158.s002.pdf]

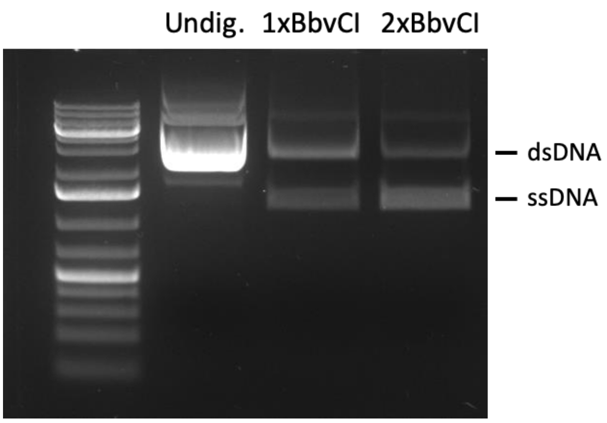

Supplementary Figure 2. Two BbvCI sites improves digestion efficiency
Plasmids with either one or two BbvCI sites were digested with Nt.BbvCI, exonuclease I and exonuclease III, per the original nicking protocol, and digestion products were visualized on an agarose gel.
